# Fabrication of Vacuum-Sealed Capacitive Micromachined Ultrasonic Transducer Arrays Using Glass Reflow Process

**DOI:** 10.3390/mi7050076

**Published:** 2016-04-25

**Authors:** Nguyen Van Toan, Shim Hahng, Yunheub Song, Takahito Ono

**Affiliations:** 1 Microsystem Integration Center (μSIC), Tohoku University, Sendai 980-8579, Japan; 2 Department of Electronic Engineering, Hanyang University, Seoul 04763, Korea; yhsong2008@hanyang.ac.kr; 3 Graduate School of Engineering, Tohoku University, Sendai 980-8579, Japan; hahng.shim@nme.mech.tohoku.ac.jp (S.H.); ono@nme.mech.tohoku.ac.jp (T.O.)

**Keywords:** capacitive micromachined ultrasonic transducer, glass reflow process, anodic bonding, medical imaging, non-destructive measurement, chemical sensing

## Abstract

This paper presents a process for the fabrication of vacuum-sealed capacitive micromachined ultrasonic transducer (CMUT) arrays using glass reflow and anodic bonding techniques. Silicon through-wafer interconnects have been investigated by the glass reflow process. Then, the patterned silicon-glass reflow wafer is anodically bonded to an SOI (silicon-on-insulator) wafer for the fabrication of CMUT devices. The CMUT 5 × 5 array has been successfully fabricated. The resonant frequency of the CMUT array with a one-cell radius of 100 µm and sensing gap of 3.2 µm (distance between top and bottom electrodes) is observed at 2.84 MHz. The *Q* factor is approximately 1300 at pressure of 0.01 Pa.

## 1. Introduction

Capacitive micromachined ultrasonic transducers (CMUTs) have a wide range of promising applications such as medical imaging [1], non-destructive measurement [2], and chemical sensing [3]. Generally, the CMUTs were fabricated using a sacrificial release method [4,5], in which the sensing gaps are formed by the selective removal of the sacrificial layer using an appropriate etchant. However, this method requires good control over the uniformity, thickness and mechanical properties of deposited films that may affect CMUT parameters such as the sensing gap height, the membrane thickness, and the residual stress. Moreover, the removal of the sacrificial layer induces the stiction of the top and bottom electrodes, especially when the sensing gap is small. A promising technique to overcome the limitation of the sacrificial release process is a fusion bonding technique investigated in [6]. The sensing gap height can be defined by the thermal oxidation layer. A nano sensing gap is possible; however, it makes the breakdown voltage decrease and the parasitic capacitance in the area between the individual cells increase. Moreover, this process requires very flat surfaces and a high temperature process (over 1100 °C). The recent process using silicon-on-insulator (SOI) wafers and anodic bonding to borosilicate glass has been reported in [7]. A single-cell array as well as one-dimensional (1D) and two-dimensional (2D) arrays with isolation trenches have been successfully demonstrated, but its cavity is not vacuum-sealed. It may make the CMUT device susceptible to liquid environments. 

The glass reflow process is a potential fabrication method for a wide range of microsystem applications [8,9,10]. A glass in silicon reflow process for three-dimensional (3D) microsystems has been presented in its simplest form [8], followed by variations to introduce additional features. A vacuum-sealed capacitive pressure sensor, microresonators and 3D microsystems have been investigated in [8]. The optical window with and without liquid penetration for an application of optical modulators is demonstrated in [9]. The enhancement of the electro-hydrodynamic printing with a high aspect ratio nozzle using glass reflow is presented in [10]. The glass reflow process uses well-known techniques such as deep reactive ion etching (RIE), anodic bonding, and annealing (high temperature treatment) to create a generic structure wafer consisting of both silicon and glass. The silicon is patterned by photolithography and deep RIE, and hermetically sealed by anodic bonding with a glass substrate. Then, the glass reflow process is performed under a high temperature process. 

In this work, CMUT arrays have been fabricated by using glass reflow and anodic bonding techniques. The silicon through–glass wafer interconnects have been fabricated by glass reflow. The anodic bonding of the silicon-glass reflow wafer with the SOI wafer is performed. Then, the handle and buried oxide layers are removed to release CMUT membranes. Finally, the electrical connections and pads are formed.

## 2. Device Structure and Working Principle

**Figure 1:**
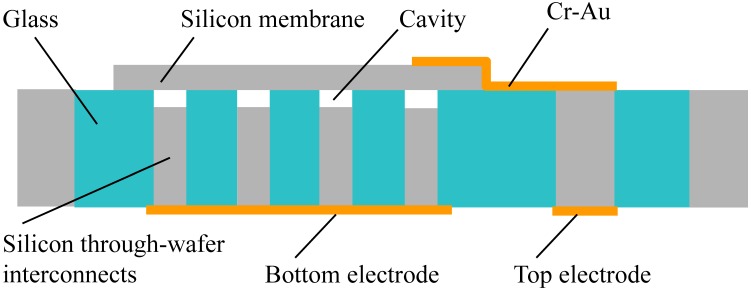
Device structure.

A schematic diagram of the CMUT array is shown in Figure 1. It consists of silicon through-wafer interconnects (bottom electrode) and thin silicon movable membranes (top electrode) suspended over a vacuum gap. The CMUT cells are isolated by the Tempax glass and Cr-Au layers are used for electrical connections and pads. The summarized parameters of the CMUT array are shown in Table 1. A circular membrane is chosen for the CMUT device with a radius of 100 μm. The maximum deflection occurs at the membrane center when a uniform pressure is applied on the entire membrane surface. The maximum displacement *x*_max_ and resonant frequency *f*_0_ of the membrane can be calculated by the equations below [11,12,13]:

(1) $$x_{\text{max}}=\frac{3\left(1-\nu^{2}\right)Pr^{4}}{16Et^{3}}$$

(2) $$f_{0}=\frac{1}{2\pi }\sqrt{\frac{k_{\text{eff}}}{m_{\text{eff}}}}$$

where *ν* is the Poisson constant of the silicon material, *P* is the applied pressure caused by the electrostatic force, *r* and *t* are the radius and thickness of the membrane, respectively, *k*_eff_ and *m*_eff_ are the effective spring constant and mass, respectively.

The mechanical stiffness (*k*_m_) and effective mass of the circular silicon membrane are shown in [14] as follows:

(3) $$k_{m}=\frac{16\pi Et^{3}}{\left(1-\nu^{2}\right)r^{2}}$$

(4) $$m_{\text{eff}}=1.84\pi \rho tr^{2}$$

where *E* is the Young’s modulus and ρ is the density of the silicon material.

The effect of electrical stiffness (*k*_e_) [15] caused by the polarization voltage (*V*_DC_) on the resonant frequency is given by Equation (5).

(5) $$k_{e}=-\frac{\varepsilon_{0}V_{\text{DC}}^{2}\pi r^{2}}{2g^{3}}$$

where ε_0_ is the electric constant ε_0_ = 8.854 × 10^−12^ Fm^−1^ and *g* is the sensing gap (distance between membrane and bottom electrode).

The resonant frequency of the membrane can be written:

(6) $$f_{0}=\frac{1}{2\pi }\sqrt{\frac{k_{\text{eff}}}{m_{\text{eff}}}}=\;\frac{1}{2\pi }\sqrt{\frac{k_{m}+k_{e}}{m_{\text{eff}}}}=\frac{1}{2\pi }\sqrt{\frac{\frac{16Et^{3}}{\left(1-\nu^{2}\right)r^{2}}-\frac{\varepsilon_{0}V_{\text{DC}}^{2}r^{2}}{2g^{3}}}{1.84\rho tr^{2}}}$$

The resonant frequency is mainly defined by its thickness and radius. For the circular membrane with a radius of 100 μm and thickness of 7 μm, the resonant frequency is estimated to be around 2.88 MHz, as shown in Table 1.

The CMUT works as a capacitor cell. When a DC voltage is applied between two electrodes, the silicon membrane is attracted toward the bottom electrode by electrostatic force. If the AC voltage is superimposed over the DC voltage, the silicon membrane will vibrate in response to the RF (radio frequency) signal and generates ultrasound. It acts as a transmitter in this case. Otherwise, if the membrane is subjected to ultrasound pressure, the electrical current is created due to the capacitance changes, and in this mode it works as a receiver.

**Figure 2:**
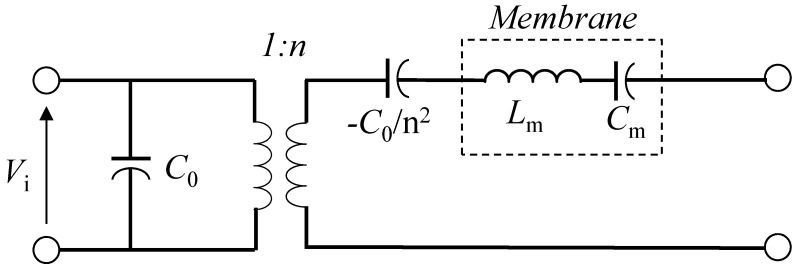
Electrical equivalent circuit model of CMUT.

The electrical equivalent circuit model of CMUTs is introduced in [4,14,16] as shown in Figure 2. It consists of the capacitance *C*_0_ of the membrane, electromechanical conversion *n* and a series inductance *L*_m_ and capacitance *C*_m_.

The capacitance *C*_0_ of the membrane is calculated using the following expression:

(7) $$C_{0}\;=\frac{\varepsilon_{0}A}{g-x}$$

where *A* is the electrode area and *x* is the membrane displacement.

*n* is one of the most important elements of the equivalent circuit. It represents the electromechanical conversion between the electrical and mechanical domain which is derived as:

(8) $$n=\frac{\varepsilon_{0}A}{{\left(g-x\right)}^{2}}V_{i}$$

There are many ways to increase the transformation ratio, such as increasing the applied voltage *V_i_*, increasing the overlap area of capacitance, or decreasing the capacitive gap.

The membrane impedance is purely imaginary and can be represented by a series inductance-capacitance circuit. *C*_m_ and *L*_m_ represent the equivalent capacitance and inductance, respectively.

(9) $$C_{m}\;=\frac{1}{k_{m}}$$

*L*_m_ = *m*_eff_ (10)

where *k*_m_ and *m*_eff_ are the spring constant and effective mass of the membrane, respectively.

The mechanical impedance *Z*_m_ of the membrane is calculated by solving the fourth-order differential equation of motion on the membrane presented in [16].

(11) $$Z_{m}\;=j\omega \rho t\frac{ak_{1}k_{2}\left[k_{2}J_{0}\left(k_{1}a\right)I_{1}\left(k_{2}a\right)\;+\;k_{1}J_{1}\left(k_{1}a\right)I_{0}\left(k_{2}a\right)\right]}{ak_{1}k_{2}\left[k_{2}J_{0}\left(k_{1}a\right)I_{1}\left(k_{2}a\right)\;+\;k_{1}J_{1}\left(k_{1}a\right)I_{0}\left(k_{2}a\right)\right]-2\left(k_{1}^{2}-k_{2}^{2}\right)J_{1}\left(k_{1}a\right)I_{1}\left(k_{2}a\right)}$$

where *J*_0_ and *J*_1_ are Bessel functions; *I*_0_ and *I*_1_ are modified Bessel functions; ω is the radian frequency and ρ and *t* are the density and thickness of the membrane material, respectively. 

Physically reasonable boundary conditions at *r = a* are that *x* = 0, which implies that the membrane undergoes no displacement at its periphery, and ${\left(\frac{d}{dr}\right)}_{x=0}$, which implies that the membrane is perfectly flat at its periphery. 

*k*_1_ and *k*_2_ are given by the equations below:

(12) $$k_{1}\;=\sqrt{\frac{\sqrt{d^{2}+4c\omega^{2}}-d}{2c}}$$

(13) $$k_{2}\;=\sqrt{\frac{\sqrt{d^{2}+4c\omega^{2}}+d}{2c}}$$

(14) $$c=\frac{\left(E+T\right)t^{2}}{12\rho \left(1-\nu^{2}\right)}$$

(15) $$d=\frac{T}{\rho }$$

where *T* is the residual stress.

## 3. Experiments

### 3.1. Fabrication Process

**Figure 3:**
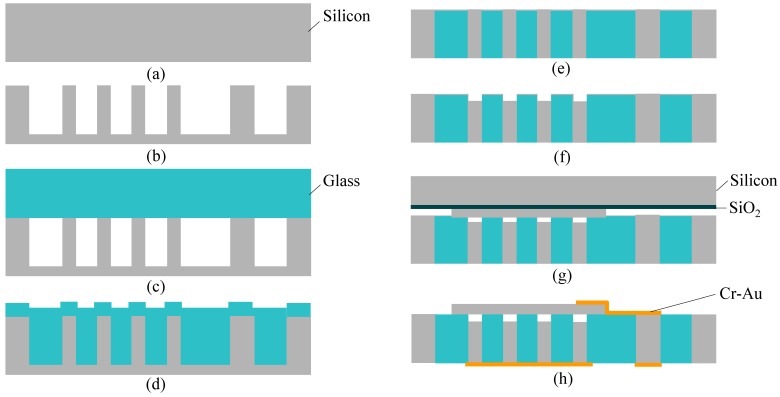
Fabrication process. (**a**) Silicon wafer; (**b**) Photolithography and deep RIE; (**c**) Anodic bonding in vacuum chamber; (**d**) Glass reflow process; (**e**) Lapping and polishing; (**f**) Photolithography and deep RIE; (**g**) Anodic bonding in vacuum chamber; (**h**) Silicon and SiO_2_ removal, electrical connection and contact pads.

Figure 3 shows the fabrication process of the CMUTs. A 300-μm-thick silicon wafer (Figure 3a) has been employed as a base. A SiO_2_ layer on the silicon wafer formed by the wet thermal oxidation with a thickness of approximately 500 nm is patterned by RIE using photoresist (OFPR 200cp) as a mask. Then, silicon is etched with a depth of around 250 μm using deep RIE by the Bosh process, forming a silicon mold (Figure 3b). The remaining SiO_2_ on silicon surfaces are removed by buffered hydrofluoric acid (BHF).

**Figure 4:**
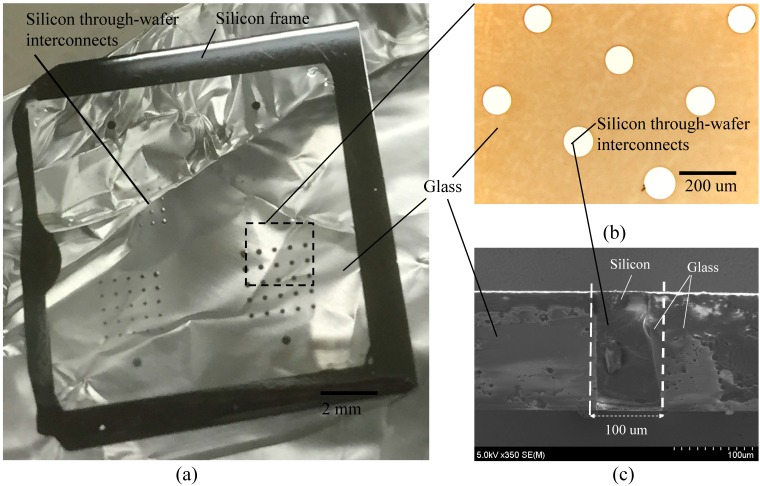
(**a**) View of 2 × 2 cm^2^ silicon-in-glass wafer; (**b**) Top view; (**c**) Cross-sectional view.

**Figure 5:**
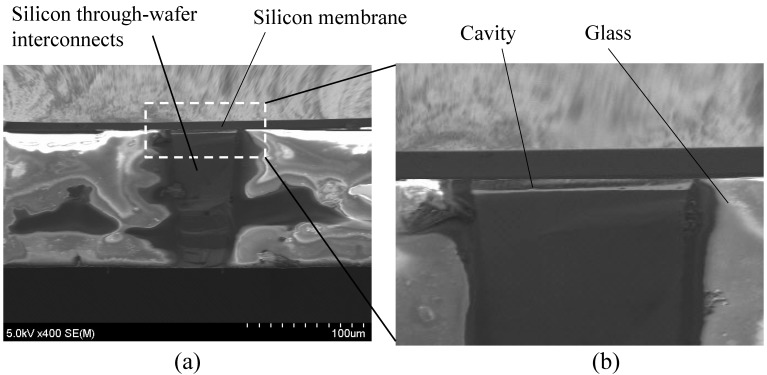
(**a**) Vacuum cavity; (**b**) Close-up image.

A -m-thick Tempax glass wafer is anodically bonded to the above silicon wafer in a high vacuum chamber (Figure 3c). The bonded silicon-glass wafer is annealed in a high temperature furnace of 750 °C for 10 h, causing the glass to fill into the silicon mold (Figure 3d). This process is called glass reflow. The above process temperature is higher than the glass transition temperature (550 °C for Tempax glass). It makes Tempax glass melt and fill into the silicon mold. After glass reflow, both sides of the silicon-glass wafer are mechanically lapped and polished by a chemical mechanical polishing (CMP) to achieve the mirror surfaces (Figure 3e). The complete filling process into silicon cavities has been investigated by optimization of the reflow conditions such as high temperature, long-running process and assistance of enhancement of the surface wettability presented in our recent research [17]. Mirror surfaces on the silicon-glass wafer have been achieved as shown in Figure 4a,b. Thus, the silicon through-wafer interconnects have been successfully fabricated (Figure 4c). Top area of the silicon through-wafer interconnect in Figure 4c is partly embraced by glass due to the cutting process using a diamond pen. So, the silicon through-wafer interconnect looks titled. A clear cross-sectional image can be achieved if the polishing process performs after diamond cutting. The high density of through-wafer interconnects is possible by an investigation on glass reflow conditions as shown in [9,17]. Next, the silicon through-wafer interconnects have been etched at the depth of approximately 3 μm for making the capacitive gaps (Figure 3f). Anodic bonding of the reflow wafer to the SOI wafer (7-μm-thick top silicon device layer, 1-μm-thick oxide layer and 300-μm-thick silicon handling layer) is performed in a vacuum chamber (Figure 3g). The handle and buried oxide layers are removed by deep RIE and RIE methods, respectively. The vacuum-sealed cavity is successfully demonstrated as shown in Figure 5a,b. Finally, the electrical connections and pads using Cr-Au layers with thicknesses of 30 nm and 300 nm, respectively, are formed by using stencil masks and a sputtering technique (Figure 3h).

### 3.2. Mesurement Setup

**Figure 6:**
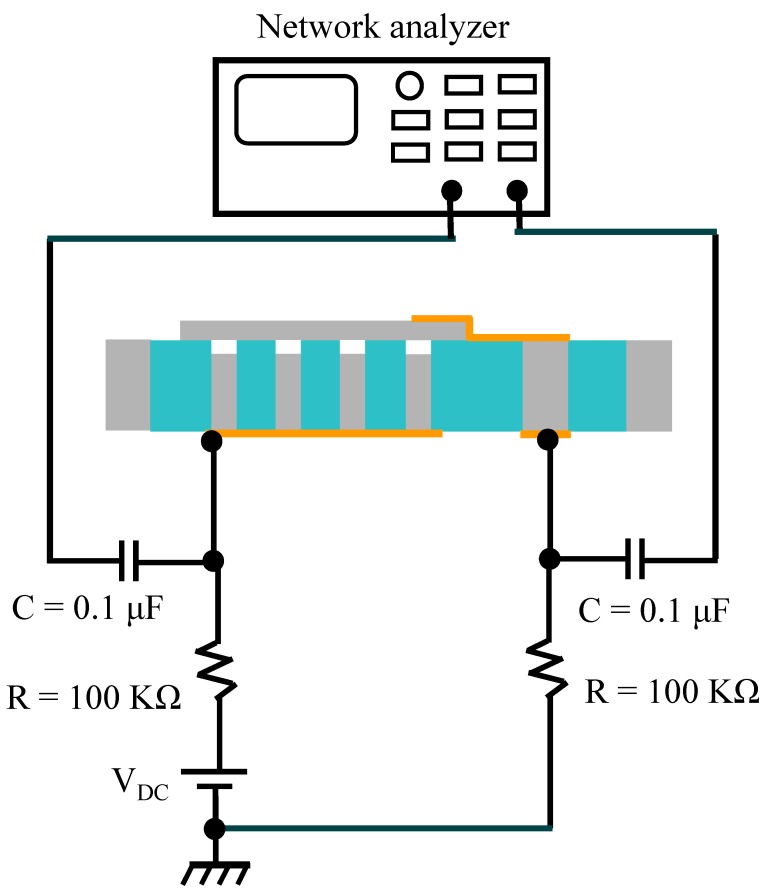
Measurement setup.

The measurement setup for the resonant characterization of CMUTs is shown in Figure 6. A network analyzer (Anritsu MS4630B, Atsugi, Japan) with a frequency range from 10 Hz to 300 MHz has been employed for this evaluation. A DC voltage is applied to the bottom electrode of CMUTs against the grounded top electrode through a 100 kΩ resistor, which decoupled from the RF output of the network analyzer using a 100 nF capacitor. The output of the device is obtained by capacitive detection between the top and the bottom electrodes. Small changes in the capacitive gap generate a voltage on the RF input of the network analyzer. The CMUT is placed inside a vacuum chamber with coaxial feed-through.

### 3.3. Measurement Results

**Figure 7:**
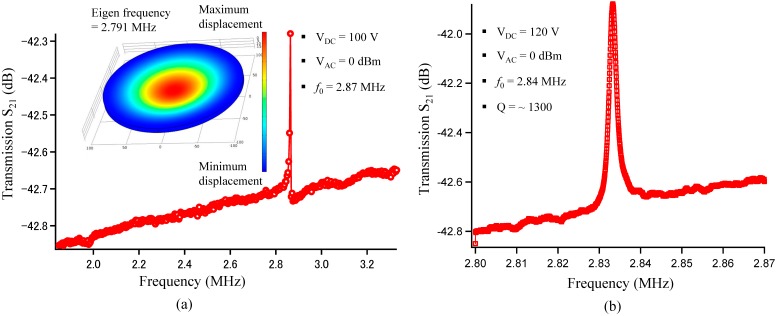
Simulation and measurement results. (**a**) Frequency response at *V*_DC_ of 100 V with wide-range observation and FEM simulation; (**b**) Frequency response at *V*_DC_ of 120 V with narrow-range observation.

The resonant characteristic of the fabricated device is evaluated, and the specifications are summarized in Table 1. Transmission *S*_21_ for the CMUT array is indicated in Figure 7. A resonant peak, which is observed under *V*_DC_ of 100 V and *V*_AC_ (alternating voltage) of 0 dBm, is found at 2.87 MHz. The resonant frequency of CMUTs mainly depends on the thickness and radius of the membrane. In this work, the membrane is uniform because the SOI wafer is employed. The thickness variation of this SOI wafer is less than 10 nm for a four-inch wafer size. The device layer on SOI is single-crystal silicon which has no stress. It means that its mechanical properties are excellent over the other deposited membranes (membranes formed by CVD, sputtering, *etc.*). Moreover, the silicon interconnects are formed by deep RIE. So, patterning silicon structures would be precise. Thus, no other resonant peaks have been observed (Figure 7a). Additionally, the simulation result (FEM—finite element method) is in good agreement with the experiment result as shown in Figure 7a.

The amplitude of the resonant frequency increases and its resonant peak shifts when changing the polarization voltage from 100 to 120 V as shown in Figure 7a,b. When the membrane is deformed from equilibrium by the electrostatic force, a restoring force will be created from the elastic spring stiffness, which acts to bring the membrane back toward equilibrium. The electrostatic force that depends on the polarization voltage is acting in the opposite direction from the elastic restoring spring force. The restoring force is effectively reduced, so the membrane acts as though it has a reduced spring constant with increasing the polarization voltage. Therefore, the resonant peak shifts to a lower frequency from 2.87 MHz to 2.84 MHz when the polarization voltage is increased from 100 V to 120 V. The effect of the electrical stiffness caused by the changing polarization voltage on the resonant frequency is explained by Equation (6) above. The *Q* factor of approximately 1300 in a vacuum environment of 0.01 Pa under measurement conditions of *V*_DC_ of 120 V and *V*_AC_ of 0 dBm is shown in Figure 7b.

## 4. Conclusions

We demonstrated the fabrication of vacuum-sealed capacitive micromachined ultrasonic transducer (CMUT) arrays using glass reflow and anodic bonding techniques. The CMUT 5 × 5 has been successfully fabricated and its resonant characteristic is evaluated. The resonant frequency of the CMUT array with a one-cell radius of 100 µm and sensing gap of 3.2 μm is found at 2.84 MHz with a *Q* factor of approximately 1300 in a vacuum environment. The proposed fabrication process is carried out by well-known techniques to create the glass compounded silicon structures. This process may be useful in fields such as opto-microfluidic devices, packaging with electrical feed-through, 3D-MEMS devices, *etc.*

## Figures and Tables

**Table 1:** Summarized parameters of CMUT array.

**Parameters**	**Values**
Membrane size (radius of membrane)	100 μm
Membrane thickness	7 μm
Array	5 × 5
Sensing gap	3.2 μm
**Applied conditions**	**Values**
Polarization voltage (*V*_DC_)	120 V
Alternating voltage (*V*_AC_)	0 dBm
Pressure level of chamber	0.01 Pa
**Resonant frequency (Calculation)**	**Value**
Resonant frequency	2.88 MHz
**FEM simulation**	**Value**
Resonant frequency	2.79 MHz
**Measurement results**	**Values**
Resonant frequency	2.84 MHz
*Q* factor	1300
